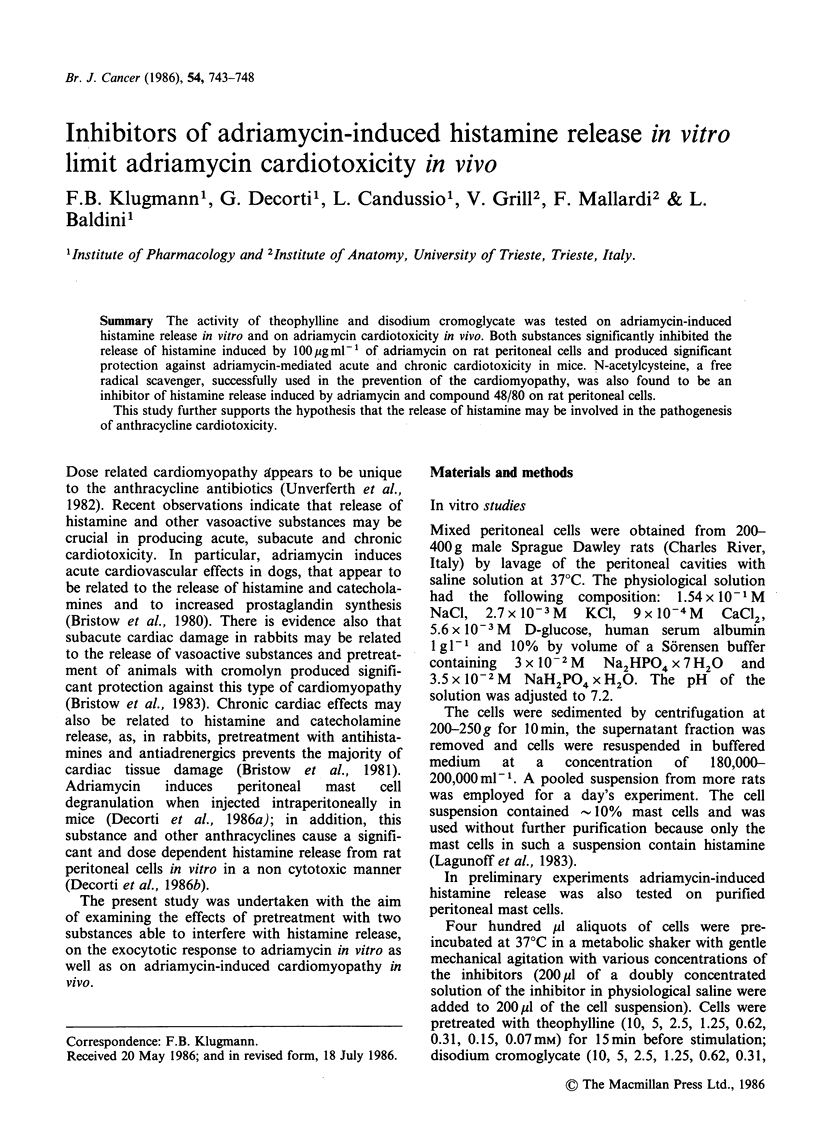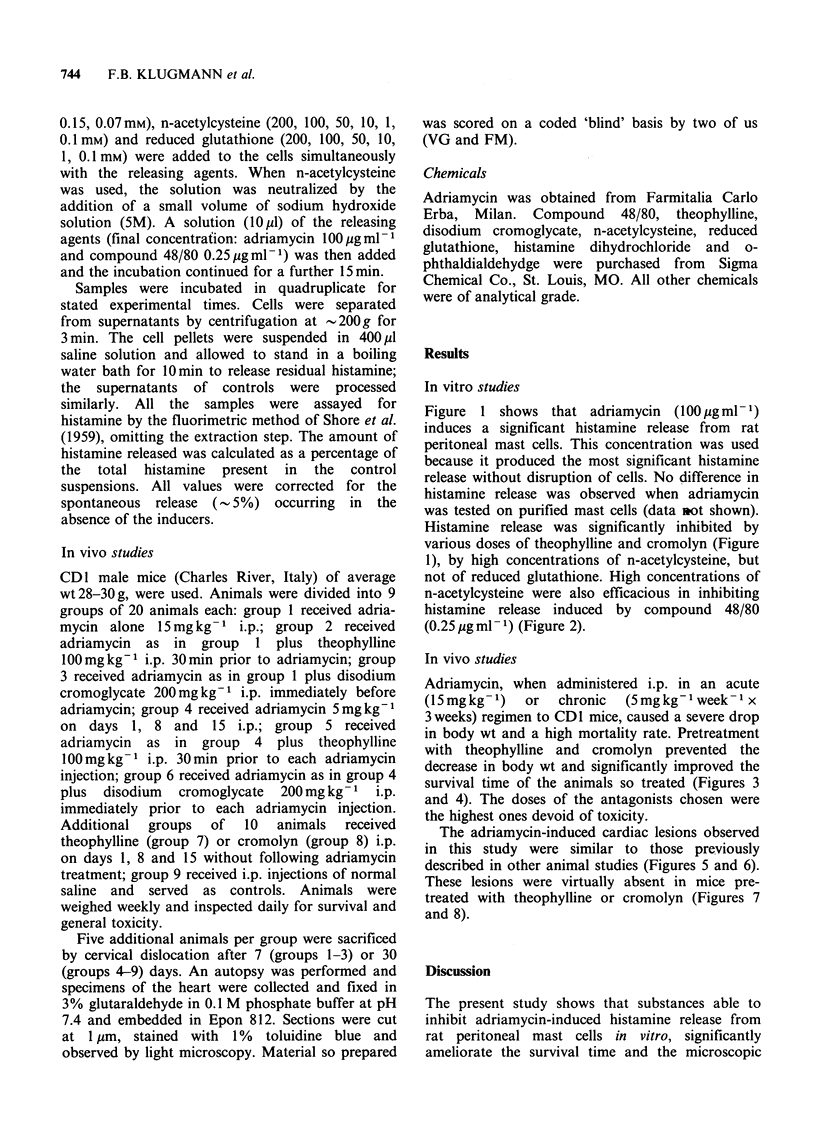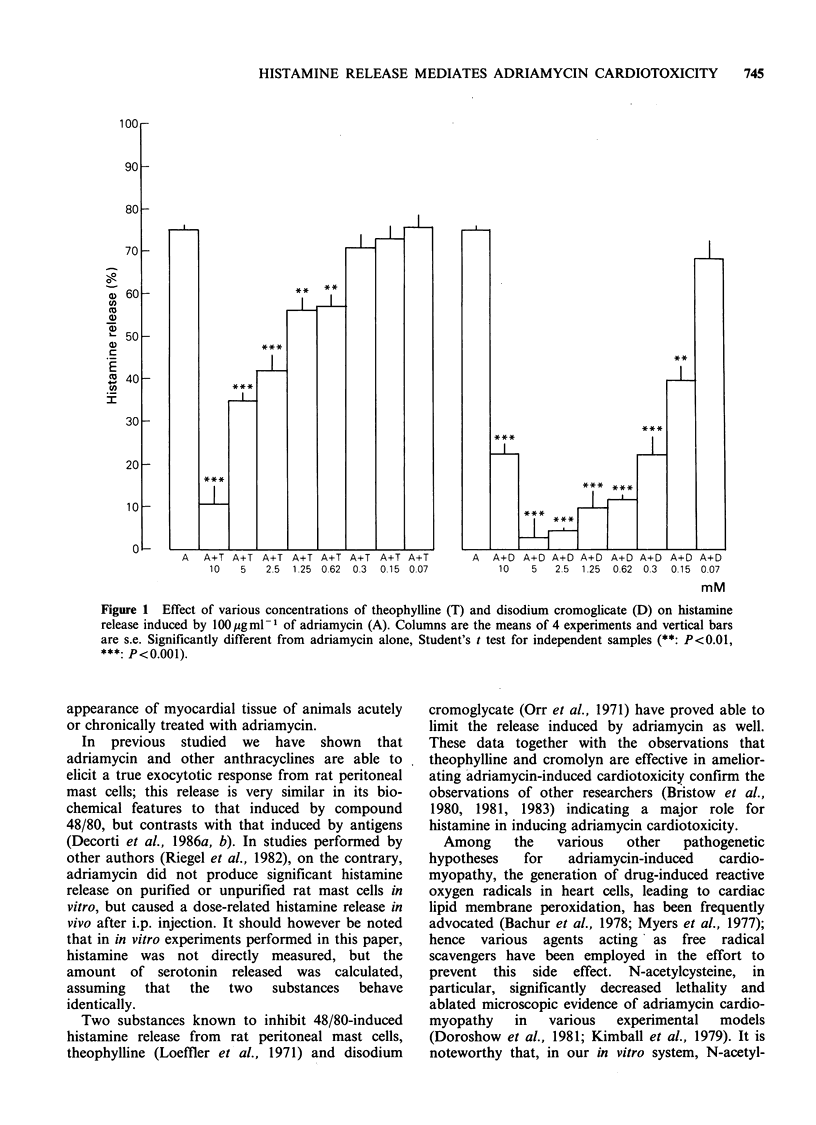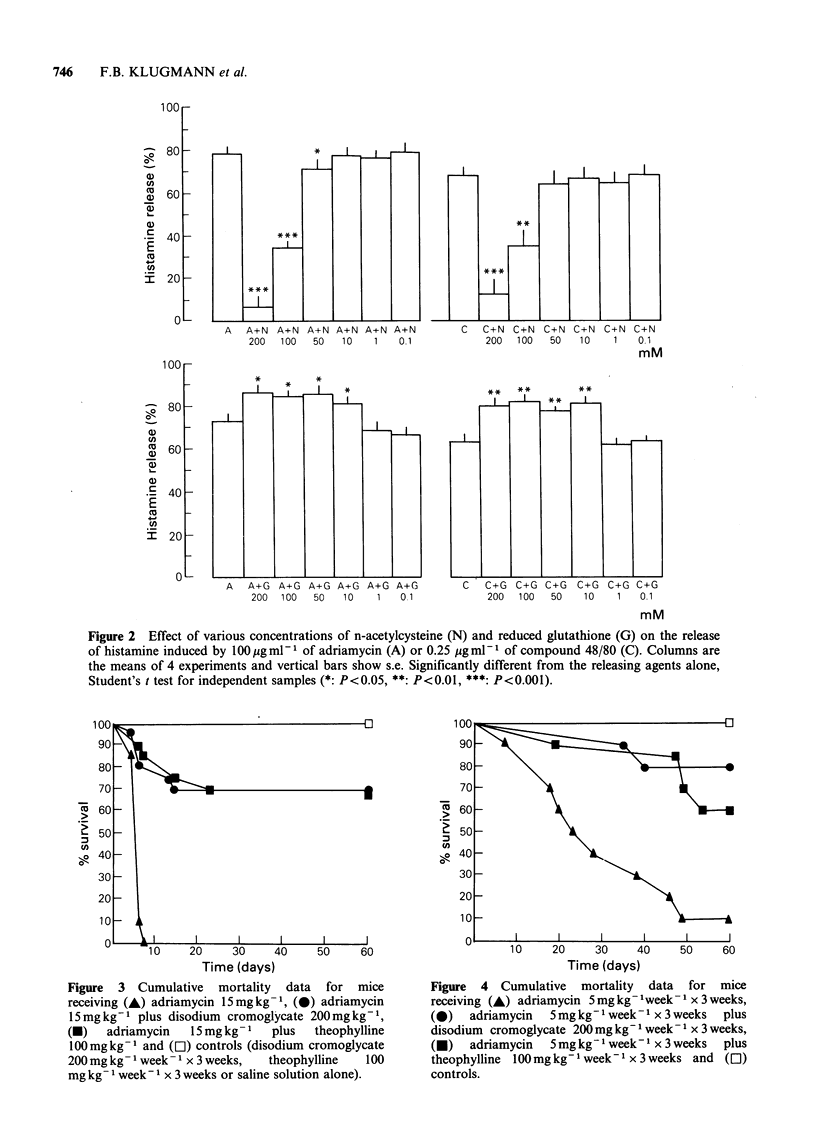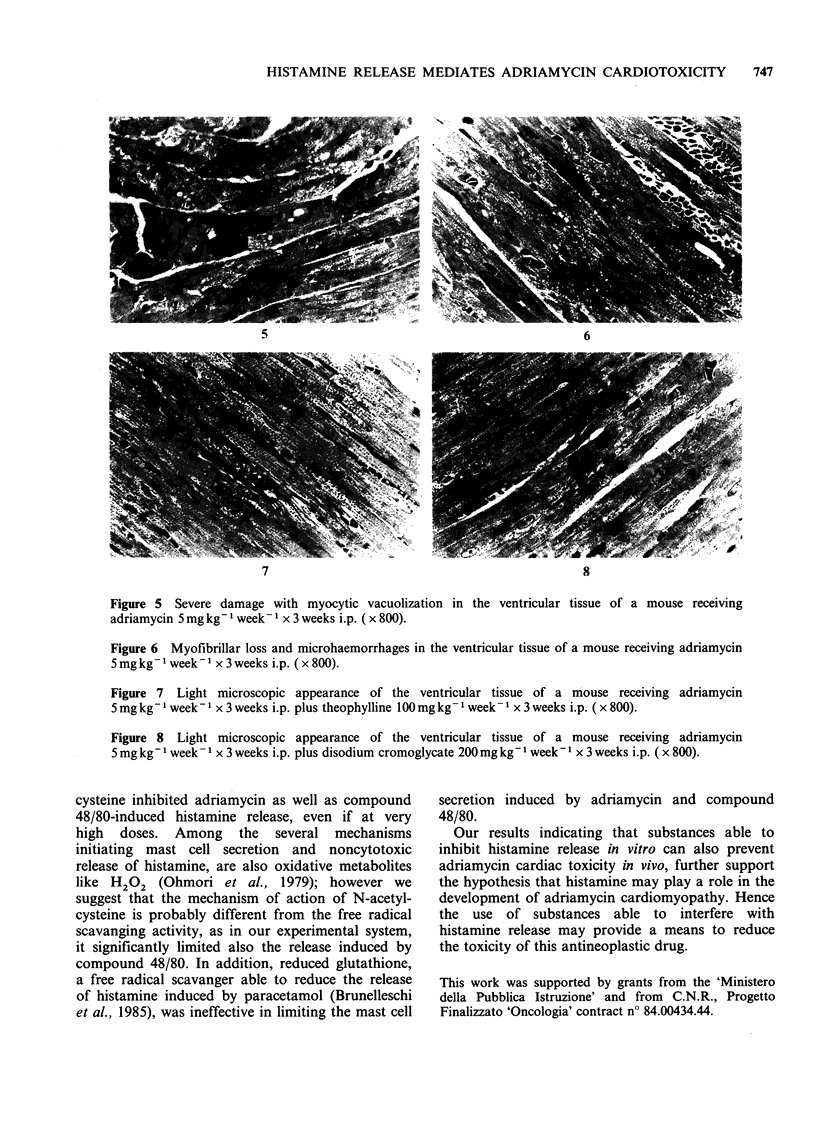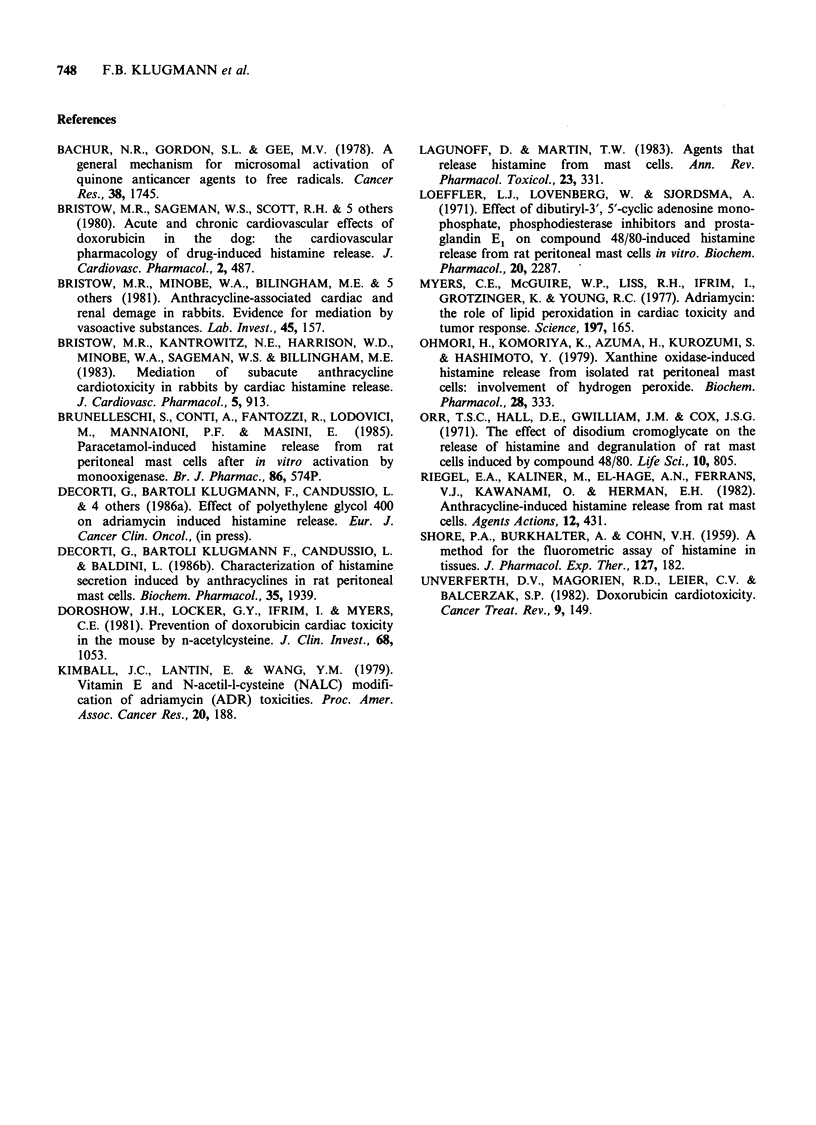# Inhibitors of adriamycin-induced histamine release in vitro limit adriamycin cardiotoxicity in vivo.

**DOI:** 10.1038/bjc.1986.235

**Published:** 1986-11

**Authors:** F. B. Klugmann, G. Decorti, L. Candussio, V. Grill, F. Mallardi, L. Baldini

## Abstract

**Images:**


*Br. J. Cancer* (1986), **54**, 743–748

### Inhibitors of adriamycin-induced histamine release *in vitro* limit adriamycin cardiotoxicity *in vivo*

F.B. Klugmann[1], G. Decorti[1], L. Candussio[1], V. Grill[2], F. Mallardi[2] & L. Baldini[1]

[1]*Institute of Pharmacology and* [2]*Institute of Anatomy, University of Trieste, Trieste, Italy.*

**Summary** The activity of theophylline and disodium cromoglycate was tested on adriamycin-induced histamine release *in vitro* and on adriamycin cardiotoxicity *in vivo*. Both substances significantly inhibited the release of histamine induced by $100\,\mu g\,ml^{-1}$ of adriamycin on rat peritoneal cells and produced significant protection against adriamycin-mediated acute and chronic cardiotoxicity in mice. N-acetylcysteine, a free radical scavenger, successfully used in the prevention of the cardiomyopathy, was also found to be an inhibitor of histamine release induced by adriamycin and compound 48/80 on rat peritoneal cells.

This study further supports the hypothesis that the release of histamine may be involved in the pathogenesis of anthracycline cardiotoxicity.

Dose related cardiomyopathy appears to be unique to the anthracycline antibiotics (Unverferth *et al.*, 1982). Recent observations indicate that release of histamine and other vasoactive substances may be crucial in producing acute, subacute and chronic cardiotoxicity. In particular, adriamycin induces acute cardiovascular effects in dogs, that appear to be related to the release of histamine and catecholamines and to increased prostaglandin synthesis (Bristow *et al.*, 1980). There is evidence also that subacute cardiac damage in rabbits may be related to the release of vasoactive substances and pretreatment of animals with cromolyn produced significant protection against this type of cardiomyopathy (Bristow *et al.*, 1983). Chronic cardiac effects may also be related to histamine and catecholamine release, as, in rabbits, pretreatment with antihistamines and antiadrenergics prevents the majority of cardiac tissue damage (Bristow *et al.*, 1981). Adriamycin induces peritoneal mast cell degranulation when injected intraperitoneally in mice (Decorti *et al.*, 1986*a)*; in addition, this substance and other anthracyclines cause a significant and dose dependent histamine release from rat peritoneal cells *in vitro* in a non cytotoxic manner (Decorti *et al.*, 1986*b*).

The present study was undertaken with the aim of examining the effects of pretreatment with two substances able to interfere with histamine release, on the exocytotic response to adriamycin *in vitro* as well as on adriamycin-induced cardiomyopathy *in vivo*.

Correspondence: F.B. Klugmann.
Received 20 May 1986; and in revised form, 18 July 1986.

### Materials and methods

### In vitro *studies*

Mixed peritoneal cells were obtained from 200–400 g male Sprague Dawley rats (Charles River, Italy) by lavage of the peritoneal cavities with saline solution at 37°C. The physiological solution had the following composition: $1.54\times10^{-1}$ M NaCl, $2.7\times10^{-3}$ M KCl, $9\times10^{-4}$ M $CaCl_2$, $5.6\times10^{-3}$ M D-glucose, human serum albumin $1\,g\,l^{-1}$ and 10% by volume of a Sörensen buffer containing $3\times10^{-2}$ M $Na_2HPO_4\times7H_2O$ and $3.5\times10^{-2}$ M $NaH_2PO_4\times H_2O$. The pH of the solution was adjusted to 7.2.

The cells were sedimented by centrifugation at 200–250 *g* for 10 min, the supernatant fraction was removed and cells were resuspended in buffered medium at a concentration of 180,000–200,000 $ml^{-1}$. A pooled suspension from more rats was employed for a day's experiment. The cell suspension contained ~10% mast cells and was used without further purification because only the mast cells in such a suspension contain histamine (Lagunoff *et al.*, 1983).

In preliminary experiments adriamycin-induced histamine release was also tested on purified peritoneal mast cells.

Four hundred $\mu l$ aliquots of cells were preincubated at 37°C in a metabolic shaker with gentle mechanical agitation with various concentrations of the inhibitors (200 $\mu l$ of a doubly concentrated solution of the inhibitor in physiological saline were added to 200 $\mu l$ of the cell suspension). Cells were pretreated with theophylline (10, 5, 2.5, 1.25, 0.62, 0.31, 0.15, 0.07 mM) for 15 min before stimulation; disodium cromoglycate (10, 5, 2.5, 1.25, 0.62, 0.31,

© The Macmillan Press Ltd., 1986

0.15, 0.07 mM), n-acetylcysteine (200, 100, 50, 10, 1, 0.1 mM) and reduced glutathione (200, 100, 50, 10, 1, 0.1 mM) were added to the cells simultaneously with the releasing agents. When n-acetylcysteine was used, the solution was neutralized by the addition of a small volume of sodium hydroxide solution (5M). A solution (10 $\mu$l) of the releasing agents (final concentration: adriamycin 100 $\mu g\,ml^{-1}$ and compound 48/80 0.25 $\mu g\,ml^{-1}$) was then added and the incubation continued for a further 15 min.

Samples were incubated in quadruplicate for stated experimental times. Cells were separated from supernatants by centrifugation at $\sim$200 *g* for 3 min. The cell pellets were suspended in 400 $\mu$l saline solution and allowed to stand in a boiling water bath for 10 min to release residual histamine; the supernatants of controls were processed similarly. All the samples were assayed for histamine by the fluorimetric method of Shore *et al.* (1959), omitting the extraction step. The amount of histamine released was calculated as a percentage of the total histamine present in the control suspensions. All values were corrected for the spontaneous release ($\sim$5%) occurring in the absence of the inducers.

### In vivo *studies*

CD1 male mice (Charles River, Italy) of average wt 28–30 g, were used. Animals were divided into 9 groups of 20 animals each: group 1 received adriamycin alone 15 $mg\,kg^{-1}$ i.p.; group 2 received adriamycin as in group 1 plus theophylline 100 $mg\,kg^{-1}$ i.p. 30 min prior to adriamycin; group 3 received adriamycin as in group 1 plus disodium cromoglycate 200 $mg\,kg^{-1}$ i.p. immediately before adriamycin; group 4 received adriamycin 5 $mg\,kg^{-1}$ on days 1, 8 and 15 i.p.; group 5 received adriamycin as in group 4 plus theophylline 100 $mg\,kg^{-1}$ i.p. 30 min prior to each adriamycin injection; group 6 received adriamycin as in group 4 plus disodium cromoglycate 200 $mg\,kg^{-1}$ i.p. immediately prior to each adriamycin injection. Additional groups of 10 animals received theophylline (group 7) or cromolyn (group 8) i.p. on days 1, 8 and 15 without following adriamycin treatment; group 9 received i.p. injections of normal saline and served as controls. Animals were weighed weekly and inspected daily for survival and general toxicity.

Five additional animals per group were sacrificed by cervical dislocation after 7 (groups 1–3) or 30 (groups 4–9) days. An autopsy was performed and specimens of the heart were collected and fixed in 3% glutaraldehyde in 0.1 M phosphate buffer at pH 7.4 and embedded in Epon 812. Sections were cut at 1 $\mu$m, stained with 1% toluidine blue and observed by light microscopy. Material so prepared was scored on a coded 'blind' basis by two of us (VG and FM).

### *Chemicals*

Adriamycin was obtained from Farmitalia Carlo Erba, Milan. Compound 48/80, theophylline, disodium cromoglycate, n-acetylcysteine, reduced glutathione, histamine dihydrochloride and o-phthaldialdehydge were purchased from Sigma Chemical Co., St. Louis, MO. All other chemicals were of analytical grade.

### Results

### In vitro *studies*

Figure 1 shows that adriamycin (100 $\mu g\,ml^{-1}$) induces a significant histamine release from rat peritoneal mast cells. This concentration was used because it produced the most significant histamine release without disruption of cells. No difference in histamine release was observed when adriamycin was tested on purified mast cells (data not shown). Histamine release was significantly inhibited by various doses of theophylline and cromolyn (Figure 1), by high concentrations of n-acetylcysteine, but not of reduced glutathione. High concentrations of n-acetylcysteine were also efficacious in inhibiting histamine release induced by compound 48/80 (0.25 $\mu g\,ml^{-1}$) (Figure 2).

### In vivo *studies*

Adriamycin, when administered i.p. in an acute (15 $mg\,kg^{-1}$) or chronic (5 $mg\,kg^{-1}\,week^{-1} \times$ 3 weeks) regimen to CD1 mice, caused a severe drop in body wt and a high mortality rate. Pretreatment with theophylline and cromolyn prevented the decrease in body wt and significantly improved the survival time of the animals so treated (Figures 3 and 4). The doses of the antagonists chosen were the highest ones devoid of toxicity.

The adriamycin-induced cardiac lesions observed in this study were similar to those previously described in other animal studies (Figures 5 and 6). These lesions were virtually absent in mice pretreated with theophylline or cromolyn (Figures 7 and 8).

### Discussion

The present study shows that substances able to inhibit adriamycin-induced histamine release from rat peritoneal mast cells *in vitro*, significantly ameliorate the survival time and the microscopic

**Figure 1** Effect of various concentrations of theophylline (T) and disodium cromoglicate (D) on histamine release induced by 100 $\mu g\,ml^{-1}$ of adriamycin (A). Columns are the means of 4 experiments and vertical bars are s.e. Significantly different from adriamycin alone, Student's *t* test for independent samples (**: $P<0.01$, ***: $P<0.001$).

appearance of myocardial tissue of animals acutely or chronically treated with adriamycin.

In previous studied we have shown that adriamycin and other anthracyclines are able to elicit a true exocytotic response from rat peritoneal mast cells; this release is very similar in its biochemical features to that induced by compound 48/80, but contrasts with that induced by antigens (Decorti *et al.*, 1986*a*, *b*). In studies performed by other authors (Riegel *et al.*, 1982), on the contrary, adriamycin did not produce significant histamine release on purified or unpurified rat mast cells *in vitro*, but caused a dose-related histamine release *in vivo* after i.p. injection. It should however be noted that in *in vitro* experiments performed in this paper, histamine was not directly measured, but the amount of serotonin released was calculated, assuming that the two substances behave identically.

Two substances known to inhibit 48/80-induced histamine release from rat peritoneal mast cells, theophylline (Loeffler *et al.*, 1971) and disodium cromoglycate (Orr *et al.*, 1971) have proved able to limit the release induced by adriamycin as well. These data together with the observations that theophylline and cromolyn are effective in ameliorating adriamycin-induced cardiotoxicity confirm the observations of other researchers (Bristow *et al.*, 1980, 1981, 1983) indicating a major role for histamine in inducing adriamycin cardiotoxicity.

Among the various other pathogenetic hypotheses for adriamycin-induced cardiomyopathy, the generation of drug-induced reactive oxygen radicals in heart cells, leading to cardiac lipid membrane peroxidation, has been frequently advocated (Bachur *et al.*, 1978; Myers *et al.*, 1977); hence various agents acting as free radical scavengers have been employed in the effort to prevent this side effect. N-acetylcysteine, in particular, significantly decreased lethality and ablated microscopic evidence of adriamycin cardiomyopathy in various experimental models (Doroshow *et al.*, 1981; Kimball *et al.*, 1979). It is noteworthy that, in our *in vitro* system, N-acetyl-

**Figure 2** Effect of various concentrations of n-acetylcysteine (N) and reduced glutathione (G) on the release of histamine induced by $100 \mu g ml^{-1}$ of adriamycin (A) or $0.25 \mu g ml^{-1}$ of compound 48/80 (C). Columns are the means of 4 experiments and vertical bars show s.e. Significantly different from the releasing agents alone, Student's $t$ test for independent samples (*: $P<0.05$, **: $P<0.01$, ***: $P<0.001$).

**Figure 3** Cumulative mortality data for mice receiving (▲) adriamycin $15 mg kg^{-1}$, (●) adriamycin $15 mg kg^{-1}$ plus disodium cromoglycate $200 mg kg^{-1}$, (■) adriamycin $15 mg kg^{-1}$ plus theophylline $100 mg kg^{-1}$ and (□) controls (disodium cromoglycate $200 mg kg^{-1} week^{-1} \times 3$ weeks, theophylline 100 $mg kg^{-1} week^{-1} \times 3$ weeks or saline solution alone).

**Figure 4** Cumulative mortality data for mice receiving (▲) adriamycin $5 mg kg^{-1} week^{-1} \times 3$ weeks, (●) adriamycin $5 mg kg^{-1} week^{-1} \times 3$ weeks plus disodium cromoglycate $200 mg kg^{-1} week^{-1} \times 3$ weeks, (■) adriamycin $5 mg kg^{-1} week^{-1} \times 3$ weeks plus theophylline $100 mg kg^{-1} week^{-1} \times 3$ weeks and (□) controls.

5

6

7

8

**Figure 5** Severe damage with myocytic vacuolization in the ventricular tissue of a mouse receiving adriamycin 5 mg $kg^{-1}$ $week^{-1}$ × 3 weeks i.p. (× 800).

**Figure 6** Myofibrillar loss and microhaemorrhages in the ventricular tissue of a mouse receiving adriamycin 5 mg $kg^{-1}$ $week^{-1}$ × 3 weeks i.p. (× 800).

**Figure 7** Light microscopic appearance of the ventricular tissue of a mouse receiving adriamycin 5 mg $kg^{-1}$ $week^{-1}$ × 3 weeks i.p. plus theophylline 100 mg $kg^{-1}$ $week^{-1}$ × 3 weeks i.p. (× 800).

**Figure 8** Light microscopic appearance of the ventricular tissue of a mouse receiving adriamycin 5 mg $kg^{-1}$ $week^{-1}$ × 3 weeks i.p. plus disodium cromoglycate 200 mg $kg^{-1}$ $week^{-1}$ × 3 weeks i.p. (× 800).

cysteine inhibited adriamycin as well as compound 48/80-induced histamine release, even if at very high doses. Among the several mechanisms initiating mast cell secretion and noncytotoxic release of histamine, are also oxidative metabolites like $H_2O_2$ (Ohmori *et al.*, 1979); however we suggest that the mechanism of action of N-acetylcysteine is probably different from the free radical scavanging activity, as in our experimental system, it significantly limited also the release induced by compound 48/80. In addition, reduced glutathione, a free radical scavanger able to reduce the release of histamine induced by paracetamol (Brunelleschi *et al.*, 1985), was ineffective in limiting the mast cell secretion induced by adriamycin and compound 48/80.

Our results indicating that substances able to inhibit histamine release *in vitro* can also prevent adriamycin cardiac toxicity *in vivo*, further support the hypothesis that histamine may play a role in the development of adriamycin cardiomyopathy. Hence the use of substances able to interfere with histamine release may provide a means to reduce the toxicity of this antineoplastic drug.

This work was supported by grants from the 'Ministero della Pubblica Istruzione' and from C.N.R., Progetto Finalizzato 'Oncologia' contract n° 84.00434.44.

**References**

BACHUR, N.R., GORDON, S.L. & GEE, M.V. (1978). A general mechanism for microsomal activation of quinone anticancer agents to free radicals. *Cancer Res.*, **38**, 1745.

BRISTOW, M.R., SAGEMAN, W.S., SCOTT, R.H. & 5 others (1980). Acute and chronic cardiovascular effects of doxorubicin in the dog: the cardiovascular pharmacology of drug-induced histamine release. *J. Cardiovasc. Pharmacol.*, **2**, 487.

BRISTOW, M.R., MINOBE, W.A., BILINGHAM, M.E. & 5 others (1981). Anthracycline-associated cardiac and renal demage in rabbits. Evidence for mediation by vasoactive substances. *Lab. Invest.*, **45**, 157.

BRISTOW, M.R., KANTROWITZ, N.E., HARRISON, W.D., MINOBE, W.A., SAGEMAN, W.S. & BILLINGHAM, M.E. (1983). Mediation of subacute anthracycline cardiotoxicity in rabbits by cardiac histamine release. *J. Cardiovasc. Pharmacol.*, **5**, 913.

BRUNELLESCHI, S., CONTI, A., FANTOZZI, R., LODOVICI, M., MANNAIONI, P.F. & MASINI, E. (1985). Paracetamol-induced histamine release from rat peritoneal mast cells after *in vitro* activation by monooxigenase. *Br. J. Pharmac.*, **86**, 574P.

DECORTI, G., BARTOLI KLUGMANN, F., CANDUSSIO, L. & 4 others (1986a). Effect of polyethylene glycol 400 on adriamycin induced histamine release. *Eur. J. Cancer Clin. Oncol.*, (in press).

DECORTI, G., BARTOLI KLUGMANN F., CANDUSSIO, L. & BALDINI, L. (1986b). Characterization of histamine secretion induced by anthracyclines in rat peritoneal mast cells. *Biochem. Pharmacol.*, **35**, 1939.

DOROSHOW, J.H., LOCKER, G.Y., IFRIM, I. & MYERS, C.E. (1981). Prevention of doxorubicin cardiac toxicity in the mouse by n-acetylcysteine. *J. Clin. Invest.*, **68**, 1053.

KIMBALL, J.C., LANTIN, E. & WANG, Y.M. (1979). Vitamin E and N-acetil-l-cysteine (NALC) modification of adriamycin (ADR) toxicities. *Proc. Amer. Assoc. Cancer Res.*, **20**, 188.

LAGUNOFF, D. & MARTIN, T.W. (1983). Agents that release histamine from mast cells. *Ann. Rev. Pharmacol. Toxicol.*, **23**, 331.

LOEFFLER, L.J., LOVENBERG, W. & SJORDSMA, A. (1971). Effect of dibutiryl-3′, 5′-cyclic adenosine monophosphate, phosphodiesterase inhibitors and prostaglandin $E_1$ on compound 48/80-induced histamine release from rat peritoneal mast cells *in vitro*. *Biochem. Pharmacol.*, **20**, 2287.

MYERS, C.E., McGUIRE, W.P., LISS, R.H., IFRIM, I., GROTZINGER, K. & YOUNG, R.C. (1977). Adriamycin: the role of lipid peroxidation in cardiac toxicity and tumor response. *Science*, **197**, 165.

OHMORI, H., KOMORIYA, K., AZUMA, H., KUROZUMI, S. & HASHIMOTO, Y. (1979). Xanthine oxidase-induced histamine release from isolated rat peritoneal mast cells: involvement of hydrogen peroxide. *Biochem. Pharmacol.*, **28**, 333.

ORR, T.S.C., HALL, D.E., GWILLIAM, J.M. & COX, J.S.G. (1971). The effect of disodium cromoglycate on the release of histamine and degranulation of rat mast cells induced by compound 48/80. *Life Sci.*, **10**, 805.

RIEGEL, E.A., KALINER, M., EL-HAGE, A.N., FERRANS, V.J., KAWANAMI, O. & HERMAN, E.H. (1982). Anthracycline-induced histamine release from rat mast cells. *Agents Actions*, **12**, 431.

SHORE, P.A., BURKHALTER, A. & COHN, V.H. (1959). A method for the fluorometric assay of histamine in tissues. *J. Pharmacol. Exp. Ther.*, **127**, 182.

UNVERFERTH, D.V., MAGORIEN, R.D., LEIER, C.V. & BALCERZAK, S.P. (1982). Doxorubicin cardiotoxicity. *Cancer Treat. Rev.*, **9**, 149.